# Draft Genome Sequence of Aeromonas lusitana sp. nov. Strain DSM 24905^T^, Isolated from a Hot Spring in Vila-Real, Portugal

**DOI:** 10.1128/genomeA.00226-18

**Published:** 2018-04-12

**Authors:** S. M. Colston, A. Navarro, A. J. Martinez-Murcia, J. Graf

**Affiliations:** a Department of Molecular and Cell Biology, University of Connecticut, Storrs, Connecticut, USA; b Genetic Analysis Strategies S.L., CEEI, Elche, Alicante, Spain; c Area de Microbiología, EPSO, Universidad Miguel Hernández, Orihuela, Alicante, Spain

## Abstract

Aeromonas lusitana sp. nov. is an isolate derived from a study aimed at characterizing Aeromonas spp. from water sources used for recreation and agricultural purposes and assessing the implications these organisms have for human and animal health. We present here the 4.52-Mbp draft genome sequence of this novel species.

## GENOME ANNOUNCEMENT

Members of the genus Aeromonas collectively occupy diverse niches ranging from free-living states in the environment to close associations with animals, sometimes causing disease in their hosts (1, 2). In the environment, Aeromonas spp. are most commonly associated with aquatic habitats, and as such, water sources are of particular interest, since they represent potential contamination routes that may impact human and animal health and disease. Some Aeromonas spp. are also implicated in opportunistic infections in humans; the majority of these have been identified as A. hydrophila, A. veronii, and A. caviae (3), although as classification of the species within this genus continues to improve, other aeromonad species, such as A. dhakensis, have also been suggested to be important infectious agents (4–6). To date, there are 31 established or proposed species of Aeromonas (7).

Aeromonas lusitana sp. nov. strain DSM 24905^T^ (=CECT 7828T, =11/6T, =MDC 2473) was isolated from a hot spring water sample from the Vila Real region of Portugal as part of a study to determine the ubiquity and diversity of Aeromonas species found in unmonitored agricultural and recreational water sources, with an emphasis on determining the antibiotic resistance and virulence factor profile of these aeromonads (8). Further characterization indicated that the isolate was likely from a novel Aeromonas species (9, 10). Genomic DNA was extracted using a thermal shock treatment and RNase A digestion and was purified by elution in DNase/RNase-free water through a silica column. Species identity was confirmed by partial sequencing of the 16S rRNA gene. Libraries were prepared using a Nextera XT kit (Illumina). The libraries were sequenced on the Illumina MiSeq platform, generating a total of 5,570,886 250-bp paired-end reads. CLC Genomics Workbench v10.1 (CLC Bio, Qiagen) was used to filter, trim, and assemble the reads *de novo*, generating 68 scaffolded contigs, with an *N*_50_ value of 148,742 and a coverage of 232×. The total genome size is 4,521,913 bp, with a G+C content of 61.0%. Automated annotation using the NCBI Prokaryotic Genome Annotation Pipeline predicted 4,046 coding sequences and 112 RNA genes. Analysis of the average nucleotide identity with that of other Aeromonas species indicated that the most closely related species is A. tecta (at 89.2%) (11).

Examining the genome of A. lusitana DSM 24905^T^ reveals the strain’s potential versatility in its metabolic capability, which includes chitin and amino sugar catabolism, an arginine deiminase pathway, and organic sulfur utilization. A. lusitana DSM 24905^T^ also encodes general secretion systems and type III and VI secretion pathways, aerolysin, and other putative toxins that may be important factors in host colonization and virulence determination (12–14). The genome contains few mobile elements and insertion sequences (IS*5*) and two loci with phage-related elements (15, 16). The addition of this genome sequence will provide insight into the phylogeny and diversity of Aeromonas species and into the determinants that result in pathogenic outcomes.

### Accession number(s).

This whole-genome shotgun project has been deposited at DDBJ/ENA/GenBank under the accession number PGCP00000000. The version described in this paper is version PGCP01000000.
